# Use, Spending, and Prices of Adalimumab Following Biosimilar Competition

**DOI:** 10.1001/jamahealthforum.2024.3964

**Published:** 2024-12-13

**Authors:** Benjamin N. Rome, Anushka Bhaskar, Aaron S. Kesselheim

**Affiliations:** 1 Program on Regulation, Therapeutics, and Law, Division of Pharmacoepidemiology and Pharmacoeconomics, Department of Medicine, Brigham and Women’s Hospital, Boston, Massachusetts; 2 Harvard Medical School, Boston, Massachusetts

## Abstract

This cross-sectional study examines the use, spending, and prices of adalimumab biosimilars in the first year of market competition in the US.

## Introduction

Adalimumab (Humira; AbbVie) is the best-selling drug in history, with more than $200 billion in global sales since its 2002 approval.^1^ In January 2023, the first biosimilar version of adalimumab became available in the US. Biosimilar competition promised improved affordability for patients and the health care system. To understand the early characteristics of this competition, we studied use, spending, and prices for adalimumab from 2022 to 2023.

## Methods

In this cross-sectional study, we examined the use of and spending on Humira and 9 adalimumab biosimilars in the 4 quarters before and after the first biosimilar entered the US market in January 2023. As the study was not human participant research, local ethics review and informed consent were not required per the Common Rule (45 CFR §46). The study followed the STROBE reporting guideline.

We measured the number of prescriptions dispensed by pharmacies from the IQVIA National Prescription Audit. US sales (net of rebates and discounts) were extracted from public manufacturer financial filings; sales data were only available for Humira and 3 biosimilar versions. We calculated the quarterly proportion of biosimilar use and mean net spending per prescription (ie, net price) using Excel, version 16.89.1 (Microsoft Corporation). We compared net price trends with Humira’s list price using data from Micromedex RED BOOK and stratified biosimilars based on those the US Food and Drug Administration (FDA) deemed interchangeable with Humira.

## Results

Total use of Humira and biosimilar adalimumab was stable at approximately 1 million prescriptions per quarter (Figure). The proportion of biosimilar prescriptions increased from 0.03% in quarter (Q)1 to 1.35% in Q4 of 2023. The single interchangeable biosimilar (adalimumab-adbm) represented 2.35% of biosimilar prescriptions in 2023. Net sales for adalimumab were $5 billion in Q4 of 2022 and decreased by 45% to $2.8 billion in Q4 of 2023. Net price per prescription declined by 43% from $5007 in Q4 of 2022 to $2837 in Q4 of 2023. In Q4 of 2023, the net price per prescription for Humira ($2798) was lower than those for biosimilars (range, $3452-$4793). In January 2023, the manufacturer increased Humira’s list price by 8%.

**Figure.**
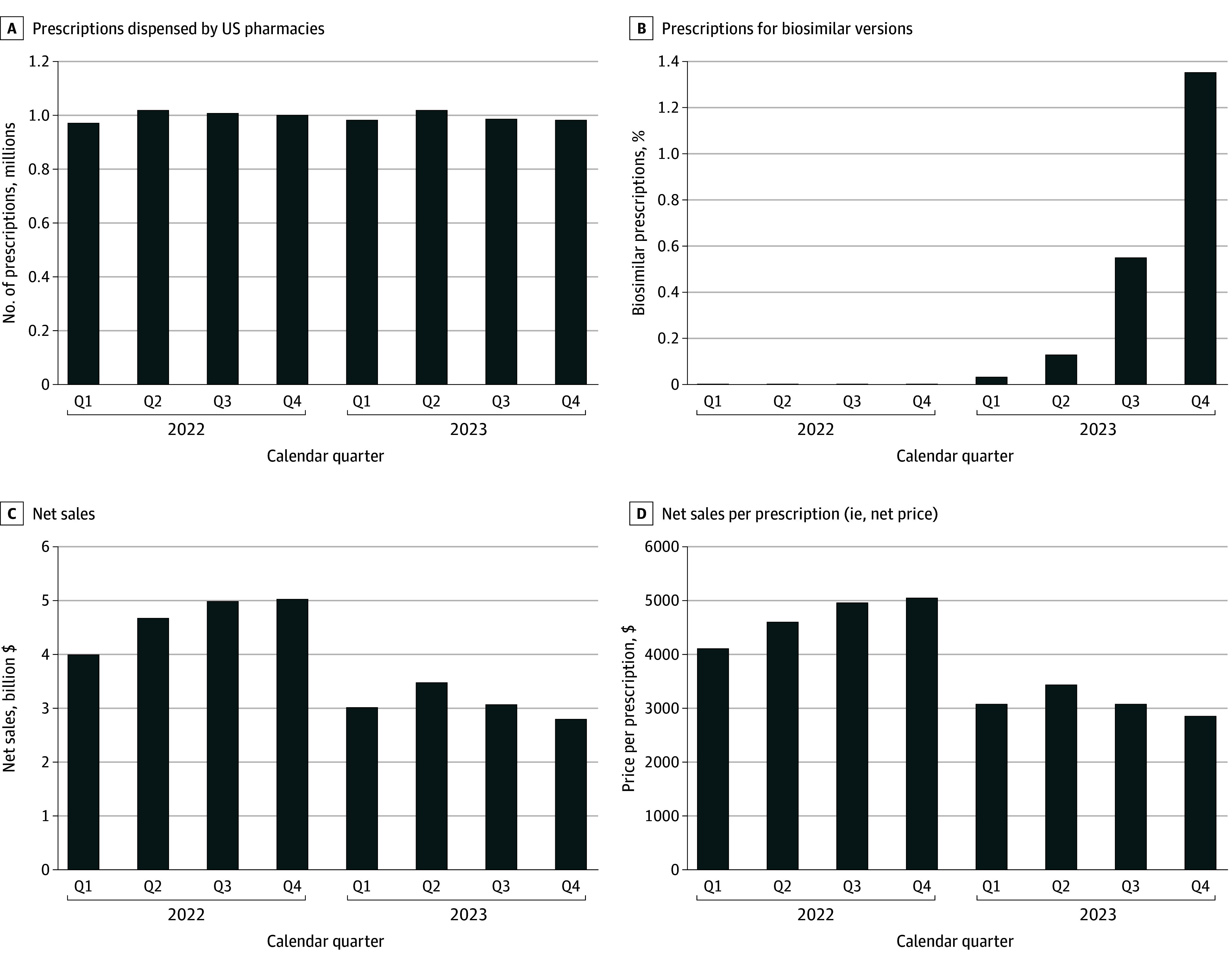
Use, Spending, and Prices of Adalimumab Before and After Biosimilar Competition Q indicates quarter.

## Discussion

In this cross-sectional study, we found that in the first year of competition, adalimumab biosimilars constituted less than 2% of prescriptions in the US. However, there was a nearly 50% decrease in adalimumab net spending and prices, likely due to rebates by AbbVie to health plans and pharmacy benefit managers for maintaining Humira’s position on formularies (list price increased while net price decreased). This study was limited by net sales excluding pharmacy and pharmacy benefit manager spending on intermediary fees and no measurement of how competition influenced patient out-of-pocket costs.

Lower health care spending is a goal of biosimilar introduction, but low uptake raises concerns that manufacturers may withdraw from the market or avoid developing future biosimilars. Compared with biosimilars, uptake of new generic drugs is often rapid, averaging 66% of the market share during the first year after brand-name market exclusivity ends.^2^ One reason is that all states allow or require pharmacists to automatically substitute generics in place of brand-name drugs; by contrast, biosimilars have additional requirements to be FDA certified as interchangeable, and state laws for biosimilar substitution are more stringent than for generics.^3^ To facilitate pharmacist substitution, Congress could eliminate the separate requirements for interchangeable biosimilars, as the FDA recommends.^4^

By January 2024, nearly all Medicare Part D plans covered Humira, while only half covered biosimilars and less than 2% preferred biosimilars over Humira.^5^ To prevent manufacturers from leveraging confidential rebates to maintain exclusive preferred formulary status, policymakers could prohibit such rebates for biologics after initiation of biosimilar competition, facilitating direct price competition and possibly lowering patient out-of-pocket costs. In January 2024, CVS Health announced removal of Humira from its standard commercial formularies in favor of a colicensed biosimilar^6^; the effects of this development on biosimilar uptake and spending should be assessed.

## Notes

Supplement.
Data Sharing Statement
