# Supplementary material for: Use, Spending, and Prices of Adalimumab Following Biosimilar Competition
Source: JAMA Health Forum. 2024 Dec 13;5(12):e243964. doi: 10.1001/jamahealthforum.2024.3964 (PMC11645644; doi:10.1001/jamahealthforum.2024.3964)
Supplement: Supplement. — Data Sharing Statement [file jamahealthforum-e243964-s001.pdf]

Data Sharing Statement

Rome. Use, Spending, and Prices of Adalimumab Following Biosimilar Competition. *JAMA Health Forum*. Published December 13, 2024. doi:10.1001/jamahealthforum.2024.3964

Data
Data available: No

Additional Information
Explanation for why data not available: Data from SSR Health and IQVIA were accessed used under a data use agreement. These data can be obtained by contacting these vendors directly.
